# Antenatal diagnosis of congenital pouch colon: a case report from the Indian subcontinent with insights into management

**DOI:** 10.1093/bjrcr/uaad005

**Published:** 2023-12-13

**Authors:** Jitendra Sharma, Rajesh Malik, Reyaz Ahmed

**Affiliations:** Department of Radiodiagnosis, AIIMS Bhopal, Bhopal, MP, 462020, India; Department of Radiodiagnosis, AIIMS Bhopal, Bhopal, MP, 462020, India; Department of Paediatric surgery, AIIMS Bhopal, Bhopal, MP, 462020, India

**Keywords:** congenital pouch colon, antenatal sonography, congenital malformation, X-ray abdomen, coloplasty, colostomy

## Abstract

Congenital pouch colon (CPC) is highly uncommon congenital anorectal malformation where a distended pouch-like structure replaces either some part of the colon or the entire colon and communicates to the genitourinary tract through a fistula. Diagnosis of CPC is usually made after birth when neonate/infant presents with abdominal distension and absence of anal opening. Making antenatal diagnosis of CPC is difficult because of the lack of specific and verifiable signs on sonography. Hence, only a few cases of antenatal diagnosis of CPC have been reported.^1^^,^^2^ In our case, CPC was suspected on a routine antenatal growth scan ultrasound in the late third trimester, showing a hypoechoic tubular-shaped lesion in the pre-sacral region. With this suspicion, we suggested an institutional delivery at a tertiary level centre, and diagnosis of type III CPC was confirmed on post-delivery imaging and emergency primary surgery, done on the day 3 of life (pouch resection, division of fistula, and protective colostomy). The child also underwent further corrective surgeries in a staged manner in second year of life and recovered completely. Beforehand diagnosis prevented any unnecessary delay in operative care, reduced postoperative complications, and improved the overall outcome of this otherwise complex condition.

## Introduction

Congenital pouch colon (CPC) is a rare condition in which a pouch-like structure replaces a variable length of the colon and communicates with the urogenital tract through a fistula.^3^ The male to female ratio ranges from 2.25:1 to 7:1.^4^ Puri et al^5^ in 1984 gave the first classification of CPC, which categorized CPC into 4 subtypes (type I to type IV) based on the severity of colon shortening and length of the proximal normal colon. Type I is the most severe form, while type IV is the least severe. Recently, Mathur et al^6^ gave a new classification, which classified CPC into 5 types (Table 1) and suggested different stage surgeries for different types.

**Table 1.** Various types of CPC, as proposed by Mathur et al.^2^^,^^6^

Types of CPCs	Classification
Type I	Absent normal colon and ileum opens into the pouch colon
Type II	Ileum opens into a normal cecum which opens into the pouch colon
Type III	Normal ascending colon and transverse colon opens into the pouch colon
Type IV	Normal colon with rectosigmoid pouch
Type V	Double pouch colon with short normal interpositioned colon segment

This report describes one antenatally detected case of anorectal malformation (ARM), a type III CPC with colo-vesical fistula and other congenital malformations such as didelphis uterus and cloaca anomaly. The CPC was suspected in routine antenatal ultrasonography (USG) with the rest of the findings discovered on postnatal investigation and during surgery.

## Clinical presentation

A 30-year-old pregnant woman with G2P1L1 status visited the Department of Radiodiagnosis for a routine sonography growth scan at 36 weeks gestation. The last sonography scans for this pregnancy, including an anomaly scan (done elsewhere), were reported as usual. Her previous child had some form of ARM, for which no documents or records were available. The rest of the obstetric history and family history was noncontributory.

## Investigations

**Figure 1.**
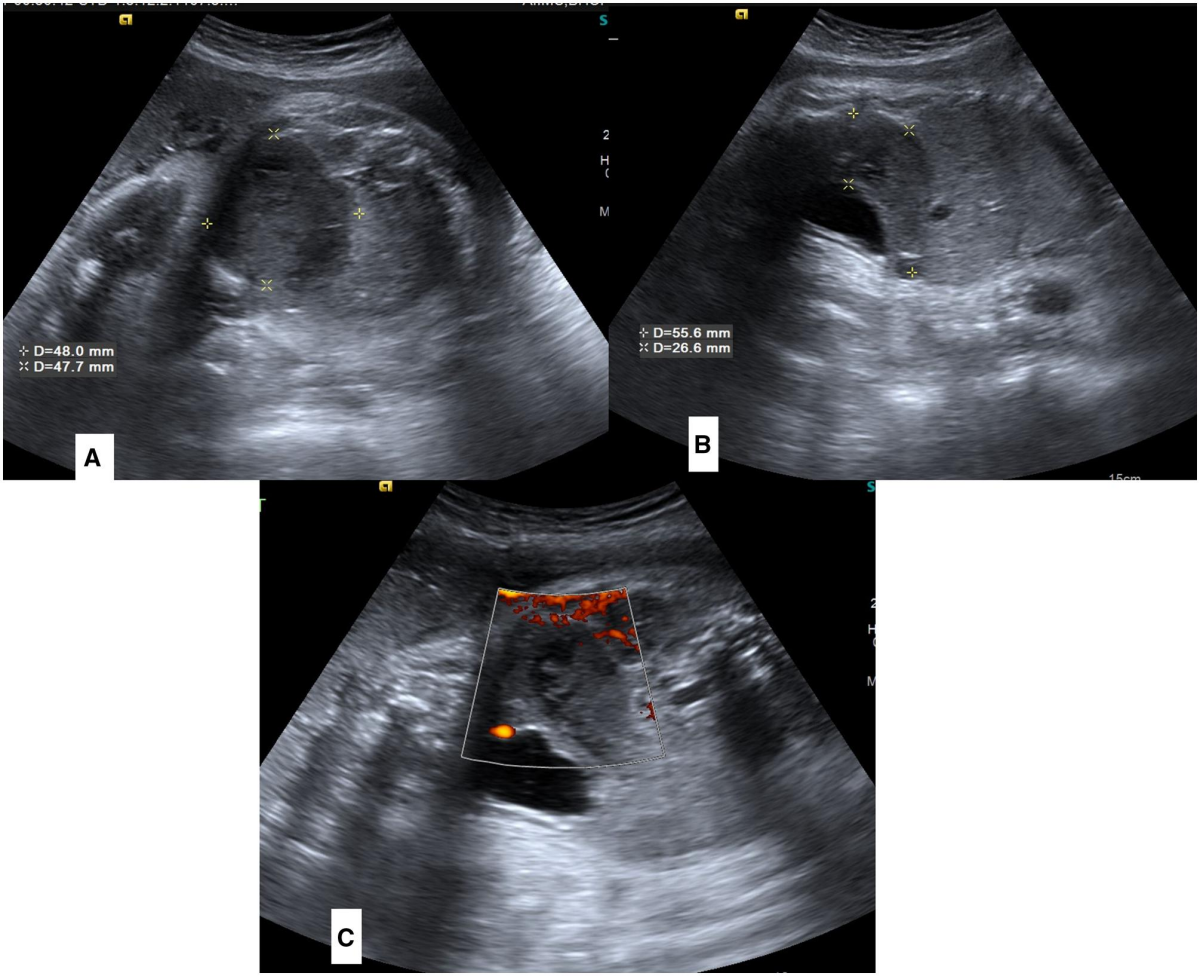
(A–C) Antenatal sonography images of foetal lower abdomen and pelvis. Sagittal (A) and Axial (B) images show a dilated pouch-like structure posterosuperior to urinary bladder (marked by asterisks). Colour doppler images (C) is showing absence of any colour flow in lesion.

Her routine growth scan ultrasound revealed a well-defined, oblong, curvilinear, hypoechoic mass in the presacral region of the foetal pelvis. This mass was posterosuperior to the urinary bladder and extended superiorly in the right side of the lower abdominal cavity (Figure 1A and B). It was almost reaching up to the right kidney and liver. However, the mass appeared separate from these organs without any mass effect. Colour Doppler application showed no blood flow within the mass (Figure 1C).

## Differential diagnosis

Considering the family history of unreported ARM in siblings and the distended pouch-like appearance of the lesion without any colour flow, we gave the possibility of CPC and a second differential of colonic duplication cyst. Because of the curvilinear shape of the lesion and the absence of colour flow, we kept the suspicion of neoplastic mass (like sacrococcygeal teratoma) low. A follow-up antenatal scan after 2 weeks also showed no remarkable changes in the lesion (images not available).

## Treatment

Based on the above suspicion, institutional delivery was planned for this pregnancy. The patient delivered a female baby at full term by standard vaginal delivery. A general examination of the baby revealed a slightly low birth weight (2.40 kg), absent anal opening, and slight abdominal distension, which progressively increased by days 2 and 3.

**Figure 2.**
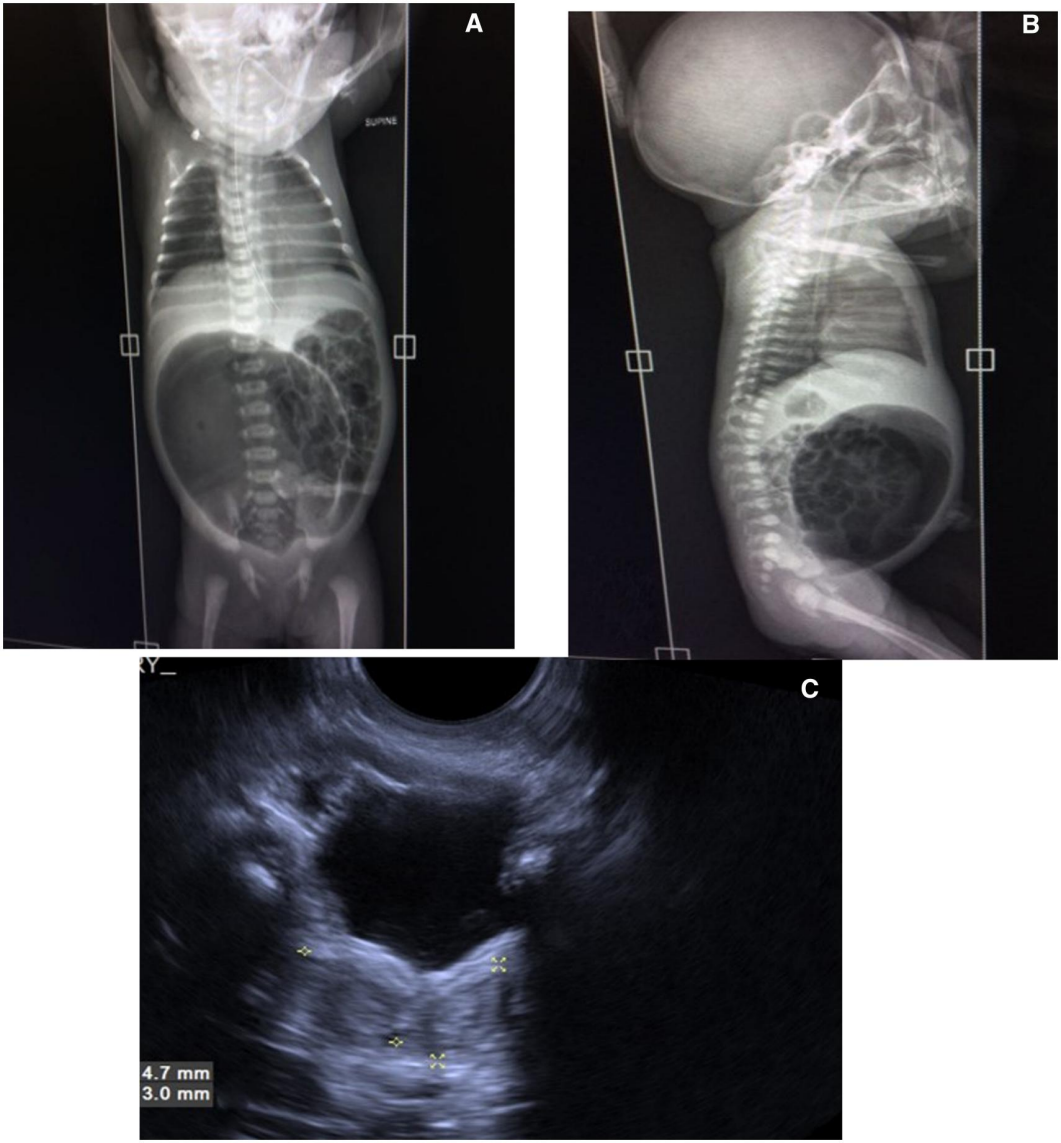
(A–C) Postnatal imaging at 24 h of life. Xray abdomen supine AP (A) and lateral (B) view showing grossly distended air-filled pouch in lower abdomen. USG scan of pelvis of baby (C) showing Didelphis uterus (marked by asterisks).

Postnatal X-ray abdomen (erect) at 24 h showed a featureless large gas shadow in the midline lower abdomen and pelvis, slightly towards the right (Figure 2A and B), confirming dilated distal large bowel.

Postnatal USG, done simultaneously, showed a bifid uterus mildly widened bladder neck; bilateral kidneys were normal (Figure 2C).

## Surgical management 

**Figure 3.**
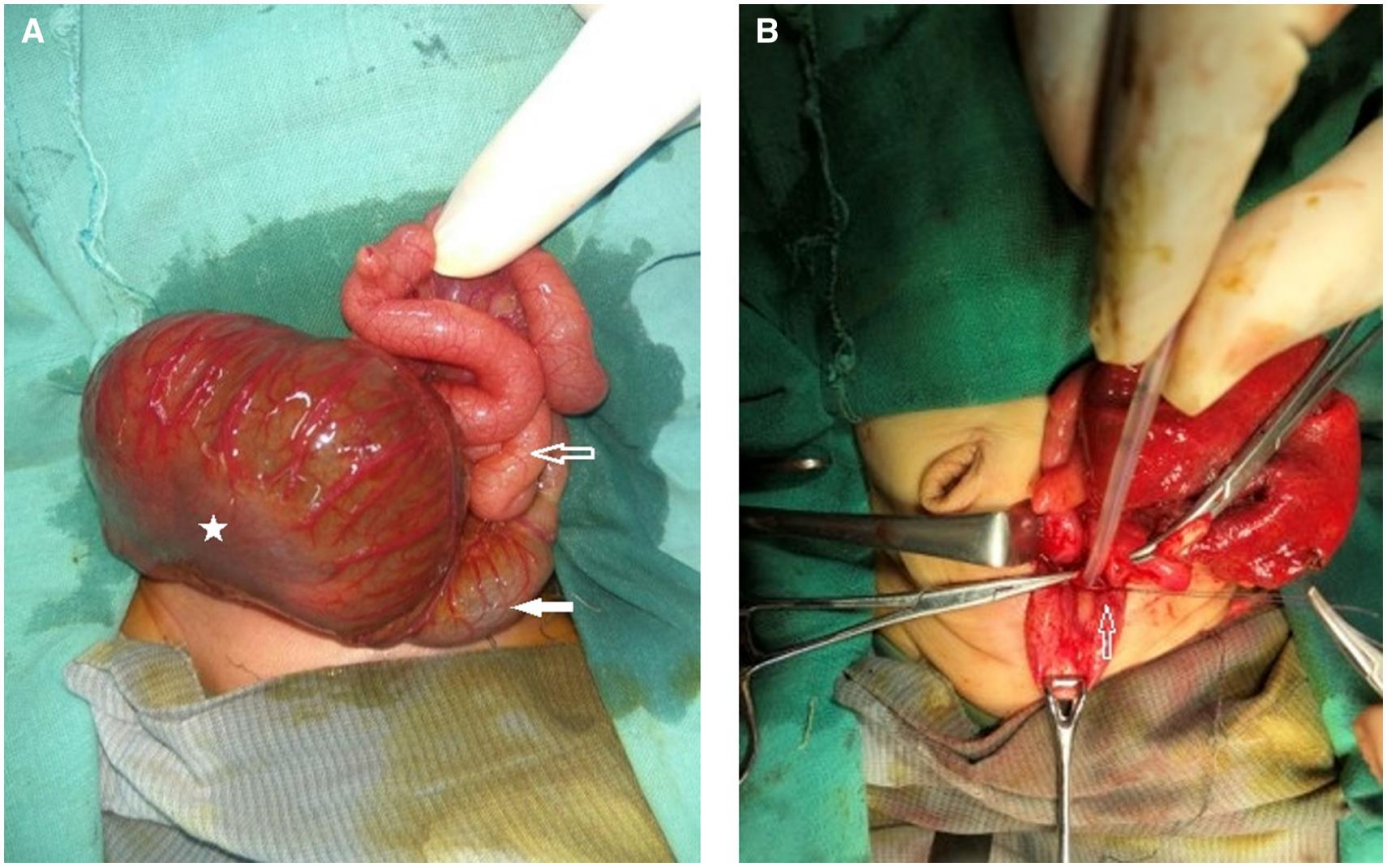
(A and B) per-operative images. (A) Dilated pouch colon (marked by star), proximal normal colon (white block arrow) and small bowel loops (hollow arrow). (B) Ligation and division of fistula (marked by hollow arrow).

Given the previous diagnosis of CPC and progressive abdominal distension, the paediatric surgeon performed surgery on the third day of life as a part of a multistage procedure. Peroperatively, a large, tensely distended pouch was found occupying the lower abdominal and pelvic cavity. Only the proximal 6 cm of large bowel was present, identified by taenia, and opened into the pouch, confirming type III CPC (Figure 3A). The terminal fistula was seen opening into a common channel between the bladder and the didelphis uterus (Figure 3B). The rest of the bowel and solid viscera were normal. The dilated pouch was excised along with ligation and division of the terminal fistula. The distalmost part of the proximal normal colon was taken out as colostomy.

## Outcome and follow-up

The child was allowed orally on postoperative day 4 and was discharged when she started feeding and gaining weight. She was kept in regular follow-up with the planning of corrective final surgery in the future. At the age of 2 years, a cystoscopy was done, which showed the presence of bladder incontinence with the absence of any common cloacal urogenital sinus. At the same time, second stage surgery of colostomy closure and abdominal-perineal pull through was done with reconstruction of neo-anus. The surgery and postsurgical period went uneventful. The child recovered well from surgery, is asymptomatic, and is regularly following up in paediatric surgery Out Patient Department (OPD).

## Discussion

This report describes the antenatal diagnosis of type III CPC in India. To the best of our knowledge, this is the first case of type III CPC diagnosed antenatally in India. CPC is an unusual abnormality associated with a pouch-like dilatation of a shortened colon with an ARM.^3^ It constitutes 6%-15% of total cases of ARM in India.^2^^,^^3^ Type 3 CPC is described as normal ascending and transverse colon opening into the dilated pouch colon, according to the classification by Mathur et al.^2^^,^^5^^,^^6^

The colonic pouch, particularly in newly born children, is thin-walled structure grossly distended with meconium and gas.^4^ However, it may be smaller and thick-walled in older children or when associated with a wide fistula. When the ileum or ascending colon enters the pouch from the right side, at a low position close to the fistula, it is referred as Type I/II CPC. Usual histopathology picture of CPC includes low number of ganglion cells with normal muscular layer in 50%, atrophic in 25% and hypertrophic in 25%.^3^

Frequently, other malformations can also be found in association with CPC: vesicoureteral reflux in 6%, intestinal malrotation in 3%, hypospadias in 3%, sacral agenesis in 3%, and Meckel’s diverticulum in 2%.^2^^,^^3^

Typical postnatal imaging finding in a prone cross-table X-ray/plain X-ray abdomen in type I or II CPC is a large gas shadow or air-fluid level on the left side of the abdomen, occupying more than half of the abdominal width and ending at high supra levator position.^4^

Coloplasty and excision have been described as surgical approaches for managing the colonic pouch.^2^^,^^3^^,^^6^ This procedure converts the colonic pouch into a long narrow tubular structure, preserves the absorptive surface of colon and improves the peristalsis and motility of the colonic pouch.^2^^,^^7^ After fistula ligation (if any present) and subtotal excision of the colonic pouch, the diameter of neo-colon should be 1.5 cm or less in the newborn.

In our case, intrauterine diagnosis had many advantages. It prepared the OBGYN team for expected delivery complications and directed the treatment according to the latest international recommendations. It also acknowledged paediatric surgeons to have a beforehand diagnosis and plan the surgery at appropriate times without any delay and in an elective, non-emergency manner.

## Learning points

- This report explains when to suspect congenital pouch colon (CPC) in antenatal USG if a presacral mass is determined and has a typical shape and contour. Since it is a fluid/meconium-filled pouch, it does not exert any mass effect over adjacent organs, nor show any blood flow on colour Doppler: A feature which can be helpful in differentiation from neoplastic lesions such as sacrococcygeal teratoma.
- CPC is a relatively common occurrence in the Asian population, particularly in the Indian subcontinent. Hence, keeping high suspicion of CPC in antenatal scans can identify pregnancies to be observed closely and planning an institutional delivery, particularly in the resource-poor situation of rural and underprivileged areas.
